# The psychological impact of human papillomavirus testing in women with borderline or mildly dyskaryotic cervical smear test results: 6-month follow-up

**DOI:** 10.1038/sj.bjc.6602411

**Published:** 2005-03-22

**Authors:** E Maissi, T M Marteau, M Hankins, S Moss, R Legood, A Gray

**Affiliations:** 1 Health Psychology Section, Psychology Department, Institute of Psychiatry, King's College London, Thomas Guy House, Guy's Campus, London SE1 9RT, UK; 2 Institute of Cancer Research, Cancer Screening Evaluation Unit, Sutton, Surrey SM2 5NG, UK; 3 Department of Public Health, Health Economics Research Centre, University of Oxford, Old Road Campus, Headington, Oxford OX3 7LF, UK

**Keywords:** human papillomavirus (HPV), anxiety, distress, smear test, quality of life

## Abstract

State anxiety (S-STAI-6), distress (GHQ-12), concern and quality of life (EuroQoL-EQ-5D) 6 months after human papillomavirus (HPV) testing in women with borderline or mildly dyskaryotic smear test results were assessed based on a prospective questionnaire study, with 6-month follow-up after the smear test result. Two centres participated in an English pilot study of HPV testing. Participants included two groups of women receiving abnormal smear test results: (tested for HPV and found to be (a) HPV positive (*n*=369) or (b) HPV negative (*n*=252)) and two groups not tested for HPV (those receiving (c) abnormal smear test results (*n*=102) or (d) normal smear test results (*n*=288)). There were no differences in anxiety, distress or health-related quality of life between the four study groups at 6 months. Levels of concern about the smear test result remained elevated in all groups receiving an abnormal smear test result, and were highest in the group untested for HPV. Predictors of concern across all groups receiving an abnormal smear test were perceived risk of developing cancer, being HPV positive or untested for HPV, sexual health worries and the smear being a woman's first smear test. The raised anxiety and distress observed in women immediately after being informed of an abnormal smear test result and that they are HPV positive was no longer evident at 6 months. Concern about the smear test result was however still raised in these women and those who tested negative for HPV, and particularly among those who did not undergo HPV testing.

There is growing interest in human papillomavirus (HPV) testing as part of the management of women with cytological abnormalities (Petry *et al*, 2003). This is reflected in the English pilot study evaluating the use of HPV testing in women with borderline or mildly dyskaryotic smear test results. However, concern has been expressed about its potential to raise anxiety beyond that commonly reported after the receipt of a borderline or mildly dyskaryotic result (McCaffery *et al*, 2002). Receipt of abnormal smear test results and referral for colposcopy are associated with high anxiety levels (Marteau *et al*, 1990; Lerman *et al*, 1991; Rogstad, 2002), some of which can be avoided by the provision of clear, salient information (Wilkinson *et al*, 1990; Marteau *et al*, 1996). We have documented that informing women that they have a borderline or mildly dyskaryotic cervical smear test result and that they are infected with HPV is associated, in the first 4 weeks of receiving such test results, with raised state anxiety, general distress and concern about the test result, above the level found in women who test negative for HPV and those not undergoing HPV testing (Maissi *et al*, 2004). We have also documented that receipt of an HPV-negative test result does not appear to reassure women receiving a borderline or mildly abnormal smear test result.

We report here the 6-month follow-up data for these women. We predicted that, in keeping with systematic review evidence, raised levels of anxiety, distress and concern will no longer be evident 6 months after the receipt of such test results (Shaw *et al*, 1999).

In addition, we report data on health-related quality of life (HRQoL). To date, most economic evaluations only report on the effectiveness of screening in terms of lives or life year gained. HRQoL data permit analysts to incorporate quality of life information directly into economic evaluations.

## MATERIALS AND METHODS

### Participants

All women who underwent a routine cervical smear test at one of two centres taking part in the English pilot study of Liquid Based Cytology (LBC) and HPV testing (2002–2003), and who received either a normal or a borderline/mildly dyskaryotic test result, were eligible for this study. They were informed of the possibility of being invited into this questionnaire-based study in the letter inviting them to attend for screening. All borderline or mildly dyskaryotic smear samples were tested for HPV. Following the end of HPV testing in the pilot, an extra group of women with such smear test results but no HPV testing was recruited to allow for the assessment of the possible reassuring effects of receiving an HPV-negative test result.

A total of 2183 women was sent a questionnaire within a week of the research team being informed that their smear test results had been sent to them. Up to two reminders were sent, within 14 and 28 days. The baseline sample of 1376 (63%) women comprised 366 women who had received a normal test result and 1010 women who had received a borderline or mildly dyskaryotic smear test result. Of this latter group, 331 were HPV negative, 536 were HPV positive and 143 were not tested for HPV.

Of the 1376 women in the initial sample, 1011 (74%) completed a second questionnaire 6 months after the receipt of their test results. The response rate varied significantly between the groups (*χ*^2^(3)=12.542, *P*=0.006), with 79% responding in the normal test result group (*n*=288), 76% in the HPV-negative group (*n*=252), 69% in the HPV-positive group (*n*=369) and 71% for the group not tested for HPV (*n*=102).

Responders at 6 months were compared with nonresponders on demographic characteristics (age, education, ethnic status, smear test history) as well as baseline measures of anxiety, distress, quality of life and concern.

Responders were significantly older (mean(s.d.)=37.5(11.6)) than nonresponders (33.5(11.1); *P*<0.001) and more likely to have had a previous smear test (91.5 *vs* 81.4%; *P*<0.001), but there were no significant differences in ethnic status or education. Comparing psychological outcomes immediately after receipt of the smear test result, responders differed from nonresponders in anxiety (mean(s.d.)=33.7(11.3) *vs* 35.9(12.0); *P*=0.003), distress (mean(s.d.)=2.3(3.1) *vs* 2.7(3.2); *P*=0.039) and concern (mean(s.d.)=4.2(2.0) *vs* 4.6(1.9); *P*=0.002), but not in HRQoL.

### Main outcome measures

*State anxiety* was assessed using the short form of the state scale of the Spielberger State-Trait Anxiety Inventory (S-STAI-6) (Marteau and Bekker, 1992), prorated to give a scale range from 20 to 80. The scores for women who had completed at least three of the six items (50%) were prorated to maximise the use of available data. The population norm for women is 35 (Spielberger *et al*, 1970). The internal reliability (Cronbach's *α*) of the scale in this study sample was 0.85 (*n*=963).

*General distress* was assessed using the 12-item General Health Questionnaire (GHQ-12) (range 1–12 with a cutoff point of 4 indicating clinical caseness (*α*=0.90, *n*=989)) (Bridges and Goldberg, 1986).

*Health-related quality of life* was assessed using the EuroQol EQ-5D questionnaire. Valuations of responders' health states were assigned using the results from a UK general population survey to estimate utility values on a scale of 0 to 1, where 1 is perfect subjective health and 0 is death (Dolan *et al*, 1995).

*Concern about the smear result* 6 months earlier was assessed using a 7-point rating scale asking women how concerned they felt about the smear test result, with higher scores indicating greater concern.

*Perceived risk of developing cervical cancer* was assessed using a 7-point rating scale assessing women's perceptions of their likelihood of developing cervical cancer in the next 10 years: higher scores indicate greater perceived risk.

*Sexual health worries* were assessed in the three study groups with borderline or mildly dyskaryotic test results using six items (range 1–5), five of which were taken from the standardised Psychosocial Effects of Abnormal Pap Smears Questionnaire (PEAPS-Q) (Bennetts *et al*, 1995). An extra item (‘Have you been worried about whether your test result would have a bad effect on your relationship with your partner?’) was added to address an issue raised by women during the initial phase of the study. Higher scores on the scale indicate greater worry (*α*=0.85, *n*=664).

*Repeat smear, HPV and colposcopic examinations:* The results of further cervical smear tests and colposcopic examinations were assessed from laboratory records after 6 months. These were used to categorise women as having either normal or abnormal smear test results at 6 months. Normal results include normal repeat smear test results, a negative HPV test result and normal colposcopy results. Abnormal results include all categories of abnormal repeat smear test results (regardless of HPV status), women who were HPV positive but had normal cytology and women with all categories of abnormal colposcopy results.

*Demographic information:* Age, highest educational qualification, ethnic background and smear history were reported at the initial assessment.

### Statistical analysis

Differences in the demographic and smear history characteristics of the four groups were assessed using ANOVA (for age, perceptions of the risk of developing cervical cancer and sexual health worries) and *χ*^2^ tests. For comparisons at baseline, ANOVA with linear trend was used with *a priori* linear contrasts after adjusting means for baseline differences in age and smear history. Since the four groups were not expected to differ at 6-month follow-up, comparisons were made using ANOVA with Tukey B *post hoc* tests to test for between-group differences where indicated. Hierarchical multiple linear regression was used to ascertain the best predictors of concern at the follow-up assessment point in the three groups of women with borderline or mildly dyskaryotic smear test results.

## RESULTS

The groups differed in age, whether the studied smear test 6 months previously was their first one or not, and whether the test was a repeat of a smear following an abnormal smear test result (Table 1). Women with borderline or mildly dyskaryotic smear test results who were HPV positive were the youngest and, related to this, it was more likely that the studied smear test was their first ever smear test, in comparison with women in the other groups.

Analysis of variance of means adjusted for age and smear history revealed that at follow-up the groups significantly differed in concern (F_3, 996_=83.39, *P*<0.001), but not in anxiety (F_3, 961_=0.40, *P*=0.752), distress (F_3, 980_=0.81, *P*=0.487) or HRQoL (F_3, 989_=0.70, *P*=0.554) (Table 2). *Post hoc* tests showed that concern was significantly lower in the ‘normal’ group and highest in the ‘HPV untested’ group, while women who were either HPV positive or HPV negative did not differ significantly in their levels of concern (Tukey B *post hoc* test, *P*<0.05).

The four groups also differed in their perceived risks of developing cervical cancer (F_3,985_=14.88, *P*<0.001). Women who were either HPV positive or untested for HPV perceived their risks of developing cervical cancer as significantly higher than women with normal smear test results and women who tested negative for HPV.

Overall, none of the three groups with abnormal test results was extremely worried about their sexual health. The group with HPV-positive test results did however score significantly higher than the other two groups (F_2,666_=30.64, *P*<0.001; Tukey B *post hoc* test, *P*<0.05).

Table 3 shows the variables entered into the hierarchical multiple linear regression to identify the predictors of concern 6 months after the initial assessment, in the three groups with borderline/mildly dyskaryotic smear test results.

Four variables independently predicted concern (*R*^2^=0.29, *P*<0.001): HPV status (not HPV tested *β*=0.17, *P*<0.001; HPV positive *β*=0.14, *P*=0.001), the studied smear having been a woman's first smear test (*β*=0.08, *P*=0.035), perceived risk of developing cervical cancer (*β*=0.43, *P*<0.001) and sexual health-related worries (*β*=0.15, *P*<0.001).

Women's comments on their questionnaires illustrating some of these predictors are provided in Box 1.

## DISCUSSION

As predicted, the receipt of a borderline or mildly dyskaryotic cervical smear test result 6 months earlier, with or without HPV testing, was no longer associated with the raised levels of state anxiety, or general distress seen within the first month of receipt of such test results. Similarly, there were no significant differences in HRQoL between the four study groups at 1 or 6 months. The raised levels of concern detected may not have had a significant impact on patient's HRQoL. Alternatively, women's HRQoL may have been affected but the EQ-5D may not have been sufficiently sensitive to detect it, as has been found in other contexts (Jenkinson *et al*, 1997).

Contrary to our predictions, concern about the test result was still raised 6 months later in women who had received an abnormal smear test result regardless of whether they had undergone HPV testing and regardless of whether they were HPV positive or negative. Concern was greatest in women who had not undergone HPV testing.

The four predictors of concern at the 6-month follow-up were HPV status, smear history, sexual health worries at follow-up and high perceived risk of developing cervical cancer. Of these, the largest predictor was perceived risk of developing cervical cancer. We did not ask women to quantify the likelihood that they could develop cervical cancer. It is therefore a hypothesis that their concern reflects an overestimate of the likelihood of developing cervical cancer given their test results. This could be tested by providing women with information on their relatively higher but absolute low levels of risk and assessing their subsequent levels of concern.

While being classified as having a normal test result at 6 months was predictive of lower concern, it explained relatively little of the variance. As can be seen from Table 1, for many women the results of repeat investigations were unavailable at the time of the 6-month follow-up. Where results were available, very few of the women who were HPV positive were subsequently classified as having normal test results.

Sexual worries were higher in women infected with HPV and were predictive of concern across the three groups receiving borderline or mildly dyskaryotic smear test results. We were unable to include a comparison group of women infected with HPV and normal smear test results as, according to the protocol of the LBC/HPV pilot, such smear samples were not further tested for HPV. Therefore, it was not possible to ascertain the concern and sexual worries that an HPV-positive result alone can lead to.

As illustrated in Box 1, many women reported trying in vain to find more information on the implications of HPV infectivity for partners. While some of this information is clearly lacking at present, it may be that providing women before testing with more of the available information about the sexually transmitted nature of HPV may better prepare them for learning that they are infected.

Women undergoing their first as opposed to a subsequent smear test are more likely to be experiencing concern 6 months after an abnormal smear test result. Neither age nor educational level was predictive. Whether this reflects no experience of a test result, normal or abnormal, or less knowledge about the screening test is unknown. It does however suggest the importance of ensuring that women undergoing their first smear test are helped to understand both the frequency and meaning of borderline/mildly dyskaryotic smear test results.

In contrast to responses within a month of receiving test results, there was some evidence 6 months later that women receiving a borderline or mildly abnormal smear test result were somewhat reassured by learning that they were HPV negative. While concern about their test results was similar to that of women who were HPV positive, nonetheless women who were HPV-negative perceived their risks of developing cervical cancer as lower and similar to those of women receiving normal test results. Although an HPV-negative result can be reassuring in terms of lowering the risk of cervical cancer, thinking of one's risk as similar to that following a normal smear test result could reflect false reassurance, which could adversely affect regular attendance for smear tests.

Borderline or mildly dyskaryotic cervical smear tests are relatively common, occurring in about 7% of cervical smear tests taken (1998/1999 figures for England). Similarly, rates of HPV infection are high, occurring in about 20% of women aged between 18 and 35 and about 7% of those aged over 50 (Cuzick *et al*, 1999). Rates of infection in those with borderline or mildly dyskaryotic smear test results are more than double these rates (unpublished report submitted to Department of Health, May 2004). Perceptions of the prevalence of a health threat affect how serious it is perceived to be, with threats perceived as more common being seen as less serious (Croyle and Jemmott, 1991). It is therefore possible that informing women of the frequency with which their test results are obtained would reduce concern. It would however be important before doing so to establish that such concern was not important in motivating women to attend for the recommended repeat smear tests or treatment. It is unknown to what extent the raised levels of concern found at 6 months are appropriate, acting to motivate women to follow recommended actions to reduce the risk of cervical cancer. We have recently found that general anxiety but not concern predicts attendance for a repeat smear test following an inadequate smear test result (unpublished data, submitted for publication). We therefore predict that raised concern is not adaptive, reflecting an overestimation of the likelihood of developing cervical cancer, which is more likely to demotivate than to motivate re-attendance. Thus, if HPV testing is incorporated in the primary care cervical screening services either for all women or only those with borderline/mild dyskaryotic cytology, clear information needs to be provided about HPV, including the absolute and relative risks of cervical cancer that HPV infection bestows.

### Limitations of the study

A total of 27% of the women who agreed to take part in the initial assessment did not complete the follow-up questionnaire reported in this paper. Those who were HPV positive who responded at the 6-month follow-up were less anxious immediately after receipt of their test results than those who did not respond. It is therefore possible that the pattern of results we report here at 6 months under-represents the anxiety, distress and concern present for these women at 6 months.

Women from ethnic minorities and those with lower levels of education were also under-represented in our sample. While we did not find that education level predicted concern, the study had insufficient power to assess the possible moderating effects of ethnicity on psychological responses to HPV testing.

### Concluding comment

The raised anxiety and distress observed in women immediately after being informed that they had an abnormal smear test result and were HPV positive is no longer evident 6 months later. Concern about the smear test result is, however, still raised in these women as well as those who received an HPV-negative test result and particularly in those who did not undergo HPV testing. Concern may be reduced by informing women of the relatively low absolute chance that they will develop cervical cancer, and that the prevalence of their particular test result is relatively high.

## Figures and Tables

**Table 1:** Demographic and clinical characteristics of the four study groups

	**Normal**	**Abnormal HPV negative**	**Abnormal HPV not tested**	**Abnormal HPV positive**		
	***n*=288**	***n*=252**	***n*=102**	***n*=369**	**Anova and *χ*^2^ tests**	***P***
Age in years mean (s.d.)	40.5 (12.1)	41.6 (11.1)	36.6 (11.1)	32.7 (9.8)	F=43.20	<0.001
College education: (% (*n*))	46.7 (128)	37.5 (90)	46.8 (44)	48.5 (172)	*χ*^2^=7.61	0.055
White ethnic background (% (*n*))	97.9 (282)	96.8 (242)	97.9 (94)	97.0 (353)	*χ*^2^=0.94	0.817
First smear test at baseline (% (*n*))	7.0 (20)	3.2 (8)	5.9 (6)	13.9 (51)	*χ*^2^=24.70	<0.001
Repeat smear result (% (*n*))						
Not available	NA	25.8 (65)	39.2 (40)	49.9 (184)		
Abnormal^a^	NA	7.9 (20)	23.5 (24)	46.1 (170)	*χ*^2^=288.99	<0.001
Normal	NA	66.3 (167)	37.3 (38)	4.1 (15)		

a Abnormal repeat smear test result includes all categories of abnormal cytology (regardless of HPV status), HPV-positive status but normal cytology and all abnormal colposcopy results.NA=not applicable.

**Table 2:** Psychological outcomes (adjusted means, s.e.) at baseline and 6-month follow-up assessments

	**Normal**	**Abnormal HPV negative**	**Abnormal HPV not tested**	**Abnormal HPV positive**	
	***n*=288**	***n*=252**	***n*=102**	***n*=369**	**F (*P*)**
State anxiety (S-STAI-6)					
Baseline	36.1 (0.7)^a^	37.5 (0.8)^b^	37.1 (1.3)^b^	38.6 (0.7)^c^	5.73 (0.017)
Follow-up	36.8 (0.8)	35.7 (0.8)	36.7 (1.3)	36.7 (0.7)	0.40 (0.752)
Emotional distress (GHQ-12)					
Baseline	2.0 (0.2)^a^	2.2 (0.2)^b^	2.1 (0.3)^b^	2.7 (0.2)^c^	8.78 (0.003)
Follow-up	2.0 (0.2)	2.0 (0.2)	1.9 (0.3)	2.3 (0.2)	0.81 (0.487)

*Concern about test result*					
Baseline	2.3 (0.1)^a^	4.6 (0.1)^b^	4.7 (0.2)^b^	5.2 (0.1)^c^	488.31 (<0.001)
Follow-up	2.0 (0.1)^a^	3.5 (0.1)^b^	4.4 (0.2)^c^	3.8 (0.1)^b^	83.39 (<0.001)
HRQoL (EQ-5D)					
Baseline	0.91 (0.02)	0.89 (0.02)	0.87 (0.02)	0.88 (0.02)	0.91 (0.340)
Follow-up	0.86 (0.02)	0.90 (0.02)	0.88 (0.04)	0.89 (0.02)	0.70 (0.554)
Perceived risk					
Baseline	3.3 (0.1)^a^	3.8 (0.2)^b^	4.3 (0.2)^b^	4.7 (0.1)^c^	55.06 (<0.001)
Follow-up	3.0 (0.2)^a^	3.3 (0.2)^a^	4.7 (0.3)^b^	4.1 (0.1)^b^	14.88 (<0.001)
Sexual health worries					
Baseline	NA	NA	NA	NA	
Follow-up	NA	1.0 (0.1)^a^	1.1 (0.1)^a^	1.8 (0.1)	30.64 (<0.001)

Values sharing the same superscripts are not significantly different (Planned linear contrasts (baseline); Tukey *post hoc* tests (follow-up)). NA=not available.

**Table 3:** Hierarchical multiple linear regression analysis predicting concern at the 6-month follow-up assessment for the three groups with borderline or mildly dyskaryotic smear test results

**Block**	***R*^2^ (*P*<0.001)**	**Variable**	***β* (*P*)**
Smear test result and history	0.039	HPV positive^a^	−0.11 (0.009)
		HPV not tested^a^	0.16 (<0.001)
		Previous smear: none^b^	0.11 (0.006)
		Previous smear: abnormal^b^	−0.01 (0.723)
Demographics	0.061	Age	−0.11 (0.007)
		Education	−0.12 (0.003)
Repeat smear	0.072	Repeat smear: normal^c^	−0.07 (0.109)
		Repeat smear: abnormal^c^	−0.02 (0.717)
Psychological	0.286	Perceived risk	0.41 (<0.001)
		Sexual health worries	0.10 (0.005)

a Reference group was HPV negative.

b Reference group was previous test normal/not known to be abnormal.

c Reference group was ‘not available’.

**Box 1:** Written comments from women

*Borderline/mildly dyskaryotic and HPV-positive smear test result*
1. My initial smear showed evidence of the HPV virus and abnormalities. I was very worried. My GP didn't have any information on this and the NHS Direct provided information that scared me (participant 490).
2. I still haven't been given any advice from my GP, practice nurse or colposcopy clinic or NHS Direct despite asking. I have since found HPV is a contributory factor in those who have cervical cancer so why is there no information available for me or my partner? (participant 304).

*Borderline/mildly dyskaryotic and HPV-negative smear test result*
1. I am still very angry that after my ‘minor changes’ result no-one could give me any statistics or guidance on how likely it would be to worsen/get better/stay the same. NHS Direct were hopeless and gave me incorrect information. I finally spoke to a doctor at the GU clinic who told me since I tested negative for HPV she was 99% sure there was no problem. It took one and a half months of worry and speaking to different people before I heard this. Surely if you test for HPV, if the result is negative one should be told what a good thing this is? (participant 469)
2. I do not consider enough information was given after repeat smear when test still showed abnormal cells. I had to visit GP for explanation of why I don't need another smear test for 5 years despite abnormal cells existing (participant 058).

*Borderline/mildly dyskaryotic smear test result, not tested for HPV*
1. I have been told for the past two years or so that my smear test has been borderline, but nothing serious, just to keep an eye on things. I feel now that this has gone on for so long that I need to speak to my GP again (participant 923).
2. Although the nurses have been good at reassuring me and answering any questions I think the repeat smear results take too long to come through and it still a nervous time (participant 919).
